# Structural Basis and Evolution of Glycan Receptor Specificities within the Polyomavirus Family

**DOI:** 10.1128/mBio.00745-20

**Published:** 2020-07-28

**Authors:** Luisa J. Ströh, Nils H. Rustmeier, Bärbel S. Blaum, Josephine Botsch, Philip Rößler, Florian Wedekink, W. Ian Lipkin, Nischay Mishra, Thilo Stehle

**Affiliations:** a Interfaculty Institute of Biochemistry, University of Tübingen, Tübingen, Germany; b Center for Infection and Immunity, Columbia University, New York, New York, USA; c Department of Pediatrics, Vanderbilt University School of Medicine, Nashville, Tennessee, USA; Cornell University; University of Pittsburgh School of Medicine

**Keywords:** polyomavirus, evolution, glycan receptor, host-pathogen interactions, sialic acid, virus tropism

## Abstract

Asymptomatic infections with polyomaviruses in humans are common, but these small viruses can cause severe diseases in immunocompromised hosts. New Jersey polyomavirus (NJPyV) was identified via a muscle biopsy in an organ transplant recipient with systemic vasculitis, myositis, and retinal blindness, and human polyomavirus 12 (HPyV12) was detected in human liver tissue. The evolutionary origins and potential diseases are not well understood for either virus. In order to define their receptor engagement strategies, we first used nuclear magnetic resonance (NMR) spectroscopy to establish that the major capsid proteins (VP1) of both viruses bind to sialic acid in solution. We then solved crystal structures of NJPyV and HPyV12 VP1 alone and in complex with sialylated glycans. NJPyV employs a novel binding site for a α2,3-linked sialic acid, whereas HPyV12 engages terminal α2,3- or α2,6-linked sialic acids in an exposed site similar to that found in *Trichodysplasia spinulosa*-associated polyomavirus (TSPyV). Gangliosides or glycoproteins, featuring in mammals usually terminal sialic acids, are therefore receptor candidates for both viruses. Structural analyses show that the sialic acid-binding site of NJPyV is conserved in chimpanzee polyomavirus (ChPyV) and that the sialic acid-binding site of HPyV12 is widely used across the entire polyomavirus family, including mammalian and avian polyomaviruses. A comparison with other polyomavirus-receptor complex structures shows that their capsids have evolved to generate several physically distinct virus-specific receptor-binding sites that can all specifically engage sialylated glycans through a limited number of contacts. Small changes in each site may have enabled host-switching events during the evolution of polyomaviruses.

## Abstract (precis)

Virus attachment to cell surface receptors is critical for productive infection. In this study, we have used a structure-based approach to investigate the cell surface recognition event for New Jersey polyomavirus (NJPyV) and human polyomavirus 12 (HPyV12). These viruses belong to the polyomavirus family, whose members target different tissues and hosts, including mammals, birds, fish, and invertebrates. Polyomaviruses are nonenveloped viruses, and the receptor-binding site is located in their capsid protein VP1. The NJPyV capsid features a novel sialic acid-binding site that is shifted in comparison to other structurally characterized polyomaviruses but shared with a closely related simian virus. In contrast, HPyV12 VP1 engages terminal sialic acids in a manner similar to the human *Trichodysplasia spinulosa*-associated polyomavirus. Our structure-based phylogenetic analysis highlights that even distantly related avian polyomaviruses possess the same exposed sialic acid-binding site. These findings complement phylogenetic models of host-virus codivergence and may also reflect past host-switching events.

## INTRODUCTION

Polyomaviruses are small double-stranded DNA (dsDNA) viruses with a circular genome of approximately 5,000 bp. More than 100 distinct polyomaviruses have been found in samples taken from diverse mammals and birds, as well as fish (1) and invertebrates (2), and the discovery of new polyomaviruses continues (3). Of these identified viruses, 14 have been proposed to be human-tropic, but only a subset has so far been directly associated with diseases in immunocompromised individuals. The JC polyomavirus (JCPyV) is the causative agent of progressive multifocal leukoencephalopathy (PML), a fatal brain disease (4), whereas BK polyomavirus (BKPyV) causes polyomavirus-associated nephropathy (PVAN) and hemorrhagic cystitis in renal transplant recipients (5). Merkel cell polyomavirus (MCPyV) plays a key role in the development of a rare form of skin cancer, Merkel cell carcinoma, and human polyomavirus 7 (HPyV7) and *Trichodysplasia spinulosa*-associated polyomavirus (TSPyV) have been linked to diseases such as thymic and lymphoid cancers and nonmalignant skin dysplasias, respectively (6–8). The recent discovery of new family members has heightened the interest in polyomaviruses and their potential contributions to human disease, in particular cancer (9–11).

To mount an infection, virions must adhere to molecules presented on the surface of the target host cell. Such molecules have been identified for many polyomaviruses, and they include gangliosides (12–15), glycoproteins (16), and glycosaminoglycans (GAGs) (17, 18). However, some polyomaviruses use other, still unknown attachment factors (19, 20). All glycan-binding polyomaviruses analyzed to date engage oligosaccharide (glycan) structures featuring terminal sialic acids that in mammals are usually presented either on gangliosides, which are molecules composed of a glycosphingolipid, i.e., a ceramide and an oligosaccharide, or on glycoproteins, which contain mostly complex and branched oligosaccharides attached to amino acids during co- and posttranslational modification steps. These sialylated glycan structures interact with loop regions at the outer margin of the major capsid protein VP1, at the apical surface of the polyomavirus capsid, as revealed by X-ray crystallography (14, 15, 21–26) and cryoelectron microscopy (27).

Sialic acids comprise a family of more than 50 naturally occurring carbohydrates that are derivatives of the nine-carbon pyranose neuraminic acid (5-amino-3,5-dideoxy-d-glycero-d-galactononulsonic acid) (28). In humans, ganglioside and glycoproteins are commonly capped with 5-*N*-acetyl neuraminic acid (Neu5Ac) in α2,3, α2,6, or α2,8 linkages (28). The Neu5Ac moiety is central for the recognition of the polyomavirus-glycan receptor. The VP1 surface exhibits an astonishing plasticity, providing virus-specific interactions for a range of Neu5Ac linkage types and further specific characteristics of Neu5Ac-containing glycans (29). Satellite residues outside a core binding pocket are often critical for establishing specificities for a single glycan receptor motif (29), Neu5Ac linkage (21), or even different sialic acid modifications (24, 30). For example, a single amino acid mutation in BKPyV VP1 is sufficient to switch the viral receptor specificity to the specificity of the related simian virus 40 (SV40) *in vitro* and in cell culture (14). A small number of mutations in the GM1 binding site of SV40 alters ganglioside usage, resulting in a change in tropism and suggesting that VP1 divergence might be driven primarily by a requirement to interact with certain receptors (31). In contrast, molecular details for interactions of polyomaviruses with the nonsialylated GAGs are still missing. GAGs are long linear polysaccharides consisting of repeating disaccharide units that are usually highly sulfated. For example, MCPyV attaches during early cell binding events to GAGs, followed by secondary interactions with a sialylated entry cofactor (17). JCPyV uses GAGs, which are also present in oligodendrocytes and astrocytes, as alternative entry pathway attachment receptors, and similarly, BKPyV virus-like particles, but not VP1 pentamers, can employ GAGs to attach to target cells (18), suggesting that GAGs bind in regions where neighboring pentamers interact within an assembled virion. A recent cryoelectron microscopy study showed additional electron density after treatment of BK virions with GAGs on top of a patch of positively charged surface in the grooves between VP1 pentamers, but the resolution of the obtained map was not sufficient to identify details of the interaction (27).

Although evidence is emerging that coreceptor or pseudoreceptor molecules as well as downstream events also significantly contribute to virus entry upon carbohydrate receptor binding (32), virus-receptor interactions are major determinants of polyomavirus host and tissue tropism (14).

New Jersey polyomavirus-2013 (NJPyV) and human polyomavirus 12 (HPyV12) are two of the most recently identified human polyomaviruses (33, 34). In both cases, a close phylogenetic relationship to animal polyomaviruses was found, raising questions about the origin and evolution of these two viruses. NJPyV was first discovered through muscle biopsy in an organ transplant recipient with systemic vasculitis, myositis, and retinal blindness (33). Histopathology analysis suggested a tropism for endothelial cells, and thus, NJPyV may have contributed to muscle and ocular damage in the patient. However, it remained unclear if the discovery reflected merely a single human transmission event (33). A recent serological study with serum samples from 706 Italians suggests seroprevalence rates of up to 57.5% in the elderly (35), while very low seroprevalence rates of about 5% in samples from 1,050 Dutch blood donors (36) and of 1.8% in Japan (37) have been detected. NJPyV is closely related by sequence to chimpanzee polyomavirus (ChPyV), and indeed, its existence had been predicted in a serological study as a potential human homolog to ChPyV prior to its discovery (38).

HPyV12 was first described in a study focusing on the identification of new human polyomaviruses in the gastrointestinal tract, spleen, and lymph nodes (34). The virus likely represents an early offshoot of a large and diverse clade comprising polyomaviruses from apes, bats, monkeys, rodents, and humans (MCPyV and TSPyV) in the initial phylogenetic analysis based on the polyomavirus large T antigen (LT) (34). However, other VP1-based analyses suggested close relationships of HPyV12 with sheep polyomavirus (ShPyV) found in commercially available sheep meat (2) and with avian viruses such as budgerigar fledgling disease polyomavirus (BFDPyV) and finch polyomavirus (FiPyV) (3, 39). More recently, new polyomaviruses possessing especially high sequence identities with HPyV12 were detected in shrews and nutria, and thus, HPyV12 may be a variant of a nonhuman polyomavirus that naturally infects these animals (40, 41). In contrast, a recent serological study with peak seroprevalence rates of 97.3% (35) indicates that HPyV12 or a related virus circulates widely in humans, outranging earlier and more recently reported seroprevalence rates of 20% (34) and 4% (36), respectively.

Regardless of their origins, the recent identification of NJPyV, HPyV12, and other polyomaviruses has raised doubts about earlier proposed evolutionary models and has triggered new phylogenetic approaches (2, 3, 42–44). In 2016, criteria for the definition of polyomavirus species based on sequences coding for LT were introduced, leading to a new classification system that has four genera: *Alpha*-, *Beta-*, *Gamma*-, and *Deltapolyomavirus* (45). Many polyomaviruses clustering in phylogenetic trees based on the whole genome or confined parts of the genome are found in distinct but closely related hosts (43), including several “human-simian pairs.” Such pairs include, for example, (i) human JCPyV and simian agent 12 (SA12) (46), (ii) human BKPyV and SV40 (46), (iii) human polyomavirus 9 (HPyV9) and African green monkey-derived lymphotropic polyomavirus (LPyV) (46, 47), and (iv) TSPyV and orangutan polyomavirus (OUPyV) (25).

A recent study suggested that host-switching events had been so far generally underestimated for dsDNA viruses and should be considered in revised evolutionary models for polyomaviruses (48). Indeed, classic reservoirs for zoonotic viruses, including primates, bats, and rodents, are highly populated by polyomaviruses, and zoonotic transmissions have been discussed for NJPyV (33) and HPyV12 (40), as well as for the most recently identified human polyomavirus, Lyon IARC polyomavirus (LIPyV), which is related to the putatively oncogenic raccoon polyomavirus (49). However, to date, the two avipolyomaviruses BFDPyV and goose hemorrhagic polyomavirus (GhPyV) and bat PyV species are the only known examples of polyomaviruses that can infect hosts belonging to multiple species and are thus capable of a host switch (50–52).

To define the structural requirements for receptor binding within the growing polyomavirus family and to connect these with the recently proposed evolutionary models, we established the glycan binding properties of the VP1 proteins of NJPyV, HPyV12, and related animal viruses. While NJPyV and HPyV12 specifically bind sialylated glycans, their binding sites differ significantly in location and architecture. NJPyV VP1 specifically engages Neu5Ac that is α2,3-linked to galactose (Gal) with a novel binding site that differs in location from the sites in all other structurally characterized sialic acid-binding polyomaviruses. Structural analyses indicate that the Neu5Ac-binding site of NJPyV is conserved in the closely related ChPyV.

In contrast, the Neu5Ac-binding site of HPyV12 is similar in location and glycan specificity to that of TSPyV (25) and three animal polyomaviruses, ShPyV, GhPyV, and FiPyV. It is therefore clear that HPyV12, TSPyV, ShPyV, GhPyV, and FiPyV (and likely additional members of the polyomavirus family) share a core glycan-binding strategy that is conserved across mammalian and nonmammalian species.

Our results expand the number of physically distinct sialic acid-binding sites in polyomaviruses and show that structure-based phylogenetic analyses may help our understanding of factors that modulate virus-carbohydrate receptor interactions, polyomavirus evolution, and potential host-switching events in the past and in the future.

## RESULTS

### Overall structures of NJPyV and HPyV12 VP1.

**FIG 1:**
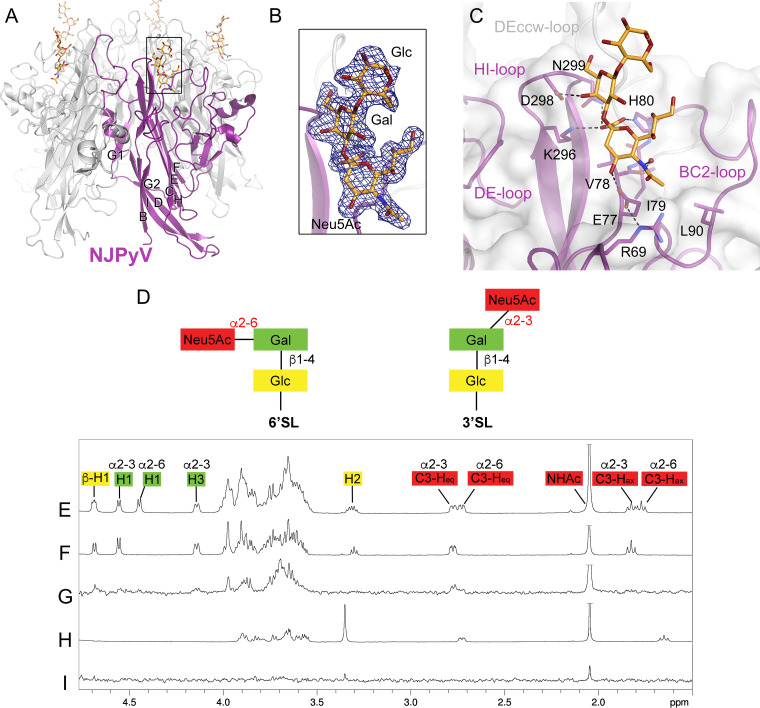
NJPyV VP1 engages the Neu5Ac-α2,3-Gal motif of 3′SL. (A) Structure of the NJPyV VP1 pentamer structure in complex with the 3′SL glycan. One VP1 monomer is highlighted in purple, the others are colored in gray. Glycans are shown in stick representation with carbons in orange, oxygens in red, and nitrogens in blue. (B) The simulated annealing difference electron density map for the 3′SL is contoured at a σ level of 2.5 and is shown with a radius of 2 Å around the ligand. (C) Interactions of NJPyV with 3′SL. Side chains of VP1 in the binding site are shown as sticks, and water molecules are shown as a red sphere. Direct and water-mediated interactions are indicated with dashed lines. (D) Schematic representation of the glycans, 3′SL and 6′SL, used for NMR experiments. Glc, glucose; Gal, galactose; Neu5Ac, *N*-5-acetyl neuraminic acid. (E) 1H NMR reference spectrum of 27 μM NJPyV VP1 with 1 mM 6′SL and 1 mM 3′SL. The equatorial H3 resonance of α2,3-linked-Neu5Ac is slightly shifted with respect to the same resonance of α2,6-Neu5Ac. (F) 1H NMR reference spectrum of 1 mM 3′SL. (G) STD NMR difference spectrum of the same sample as shown in panel D. (H) 1H NMR reference spectrum of 2 mM 2-*O*-methyl sialic acid and 27 μM NJPyV VP1. (I) STD NMR difference spectrum of the same sample as shown in panel G. Neu5Ac amide methyl group resonances are truncated.

**FIG 2:**
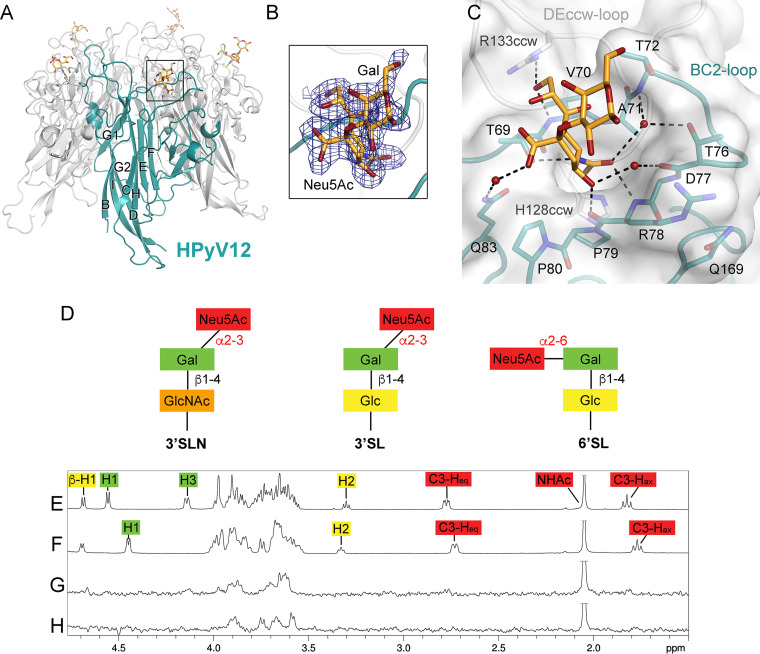
HPyV12 VP1 interacts with α2,3-linked and α2,6-linked sialic acids. (A) Structure of HPyV12 VP1 complexed with 3′SLN. One VP1 monomer of the VP1 pentamer is highlighted in deep teal. (B) A simulated annealing difference electron density map contoured at a σ level of 2.5 is displayed with a radius of 2 Å around the Neu5Ac-Gal motif of 3′SLN. (C) Close-up view of the binding site for 3′SLN. (D) Schematic representation of glycans used for X-ray structure determination (3′SLN) and NMR experiments (3′SL and 6′SL). GlcNAc, *N*-acetylglucosamine; Glc, glucose; Gal, galactose; Neu5Ac, *N*-5-acetyl neuraminic acid. (E) 1H NMR reference spectrum of 50 μM HPyV12 VP1 with 2 mM 3′SL. (F) 1H NMR reference spectrum of 50 μM HPyV12 VP1 with 2 mM 6′SL. (G) STD NMR difference spectrum recorded with the same sample as shown in panel E. (H) STD NMR difference spectrum recorded with the same sample as shown in panel F. Neu5Ac amide methyl group resonances are truncated.

In order to enable an understanding of their structural features and receptor-binding strategies, we expressed and purified VP1 pentamers of NJPyV and HPyV12 and solved X-ray structures of both native and liganded proteins (see Materials and Methods) (Table 1). The two pentamers lack N- and C-terminal residues, which renders them assembly incompetent but does not affect the structure of the VP1 surface loops and binding of sialylated ligands (53). Complexes were prepared by soaking crystals with α2,3-linked sialyllactose (3′SL) for NJPyV (Fig. 1D) and with α2,3-linked sialyllactosamine (3′SLN) for HPyV12 (Fig. 2D). Both trisaccharides feature a terminal Neu5Ac moiety and thus represent terminal oligosaccharide structures usually included in more-complex branched glycans on glycoproteins. The 3′SL glycan is, for example, also found linked to a ceramide in the context of various gangliosides. All structures were solved to high resolution, ranging from 2.3 Å to 1.55 Å, with excellent refinement statistics (Table 1). As expected, both proteins feature the conserved jelly-roll fold typical for polyomavirus VP1. This fold includes two antiparallel β-sheets, B, I, D, and G2 and C, H, E, and F (Fig. 1A and Fig. 2A), which are connected by variable surface loops named after the β-strands connected by them (12, 14, 15, 19–25, 53, 54). The outer surface of a polyomavirus is exclusively defined by the BC, DE, EF, and HI loops, and it is therefore not surprising that the two viruses engage glycans via binding sites formed by the four loops. The long BC loop emanates from the top of VP1 in two different directions, which are referred to as the BC1 and BC2 loops.

**TABLE 1:** Crystallographic data collection and refinement statistics for native and complexed NJPyV and HPyV12 VP1 structures

Parameter^*a*^	Value for indicated structure^*b*^			
	NJPyV VP1 (6Y5X)^*c*^	NJPyV VP1- 3′SL (6Y5Y)	HPyV12 VP1 (6Y5Z)	HPyV12 VP1- 3′SLN (6Y60)
Data collection statistics				
Space group	P2_1_	P2_1_	P2_1_	P2_1_2_1_2_1_
Unit cell dimensions				
*a, b, c* (Å)	86.48, 151.76, 130.91	86.38, 151.08, 130.62	61.22, 136.42, 85.38	83.32, 141.72, 251.30
β (°)	106.85	106.56	109.57	90
Resolution range (Å)	48.30–2.30 (2.36–2.30)	48.20–1.80 (1.91–1.80)	44.10–1.55 (1.64–1.55)	47.40–1.80 (1.91–1.80)
No. of unique reflections	143,046 (10,546)	295,110 (47,350)	181,696 (28,058)	274,251 (42,872)
Total no. of reflections	828,661 (60,401)	1,695,688 (255,694)	689,847 (106,706)	1,930,655 (294,756)
*R*_meas_ (%)	25.1 (104.5)	15.6 (106.4)	5.6 (45.6)	12.5 (177.2)
*I*/σ*I*	6.7 (1.6)	9.8 (1.6)	16.4 (2.9)	12.2 (1.3)
*CC*_1/2_ (%)	98.4 (63.8)	99.6 (66.6)	99.9 (85.5)	99.9 (72.5)
Completeness (%)	99.9 (99.9)	99.8 (99.3)	95.0 (90.9)	99.4 (97.0)
Wilson B-factors (Å^2^)	29.3	26.0	24.2	33.3
				
Refinement statistics				
*R*_work_/*R*_free_ (%)	18.9/24.4	15.9/19.6	14.3/16.9	20.4/24.3
No. of atoms				
Protein	21,197	21,615	10,318	19,809
Water	839	2,796	1,547	1,829
Glycan		430		287
B-factor (Å^2^)				
Protein	29.8	23.3	17.5	33.3
Water	24.4	33.4	30.4	37.5
Glycan		31.9		41.1
RMSD				
Bond length (Å)	0.008	0.010	0.009	0.009
Bond angle (°)	1.578	1.656	1.548	1.629

a *R*_meas_, data redundancy-independent R-factor; *I*/σ*I*, intensity-to-noise ratio; *CC*_1/2_, half-set correlation coefficient; *R*_work_, refinement R-factor of work set; *R*_free_, refinement R-factor of test set.

b Values in parentheses correspond to the highest data resolution shell.

c PDB accession numbers are given in parentheses after the structure designations.

### Structure of NJPyV bound to 3′SL.

NJPyV VP1 binds the α2,3-Neu5Ac-Gal motif of 3′SL in a binding site that is located between the HI and BC loops of one VP1 monomer (Fig. 1C). The DE loop, which is elongated in NJPyV VP1 compared to other structurally known polyomavirus VP1 pentamers, is not involved in any interactions with the glycan. Instead, side chains of residues K296 and N299 from the HI loop and residue H80 of the BC2 loop engage the Neu5Ac carboxylate group. Residue E77 interacts with the Neu5Ac C-4 hydroxyl, and the *N*-acetyl group forms a water-mediated hydrogen bond with the backbone of residue V78. Additionally, hydrophobic interactions between the *N*-acetyl methyl group, residue L90, and the hydrophobic part of the E77 side chain are seen, but the Neu5Ac glycerol chain is facing into solution and is not engaged by VP1. The Gal ring interacts with residue D298 via a hydrogen bond formed to the C-4 hydroxyl and via hydrophobic interactions of its C-6 atom. Like most polyomaviruses, NJPyV VP1 does not undergo any rearrangements upon glycan binding (root mean square deviation [RMSD] of 0.2 Å for the structural superposition of native and liganded NJPyV VP1 Cα atoms [55]).

### NJPyV VP1 binds specifically to α2,3-linked sialic acids in solution.

In order to confirm the observed interactions and to probe for specificity of binding, we examined the interactions of 3′SL and related glycans with NJPyV VP1 pentamers in solution using saturation transfer difference nuclear magnetic resonance (STD NMR) measurements (56, 57). This technique has been used to study similar protein-glycan interactions that are typically characterized by weak binding affinities (57, 58). It is based on the fast cross-relaxation of excited states in large molecules and the intermolecular nuclear Overhauser effect (NOE) between protons of a protein and protons of a ligand within a complex with a fast off-rate. In short, after selective excitation of proton resonances of the protein, ligand protons at distances of up to 5 Å from the protein protons receive saturation transfer from the protein during the lifetime of the complex through intermolecular NOEs and are observed in the STD NMR difference spectrum.

The STD NMR experiment showed that NJPyV VP1 pentamers specifically bind 3′SL but not 6′SL in solution (Fig. 1E to G). This is very much consistent with the crystal structure of the NJPyV-3′SL complex, which elucidated interactions with both Neu5Ac and Gal, suggesting that a defined orientation (or linkage) between the two sugars is required for the interaction. In our crystal structure, Gal forms fewer interactions than Neu5Ac and contributes only about 30% of the total interface area buried upon 3′SL binding. Likewise, in the NMR spectrum, only resonances belonging to the Neu5Ac moiety of 3′SL are observed, and the well-dispersed resonances for protons H1 and H3 of the Gal or protons of the terminal Glc are not visible in the STD NMR difference spectrum. The interactions of α2,3-linked Gal with VP1 nevertheless appear critical for ligand recognition, as isolated sialic acid, represented by the compound 2-*O*-methyl-αNeu5Ac, clearly did not interact with NJPyV VP1 in solution (Fig. 1H and I).

### Structure of HPyV12 bound to 3′SLN.

In the crystal structure of the HPyV12 VP1 pentamer in complex with 3′SLN, the Neu5Ac moiety of the linear trisaccharide is engaged in a binding site mainly formed by the BC2 loop of one VP1 monomer (Fig. 2A to C). The Neu5Ac *N*-acetyl group inserts into a shallow cavity, forming a hydrogen bond between its amide group and residue T69. Additional, nonpolar interactions involve the methyl group and residues P79 and H128 of the counterclockwise (ccw) VP1 monomer. Finally, the *N*-acetyl carbonyl oxygen forms direct and water-mediated hydrogen bonds with the backbone amide groups of R78 and T72, respectively. The Neu5Ac glycerol chain forms hydrogen bonds with the side chain of R133 from the ccw DE loop and with the backbone amine of residue V70. On the other side of the BC2 loop, the Neu5Ac C-4 hydroxyl group interacts with the protein backbone. The Neu5Ac carboxylate group is not involved in any direct interactions with VP1, only forming a water-mediated hydrogen bond with the side chain of residue Q83. Cα atoms of the BC2 loop (residues 67 to 82) from the liganded HPyV12 VP1 structure superpose with the respective residues of the native structure with an RMSD of 0.3 Å (55), indicating that the Neu5Ac docks into a preformed binding pocket within the BC2 loop and that no conformational changes occur upon ligand binding. Well-defined electron density was observed for the Neu5Ac ring in all 10 VP1 binding sites of the asymmetric unit, and in some sites the Gal ring could also be unambiguously built into the electron density. Although the Gal does not interact with VP1 via directed polar interactions, it is likely that conformational restraints of the Neu5Ac-Gal glycosidic bond in the bound state stabilize the Gal ring in a preferred orientation.

### HPyV12 VP1 interacts with α2,3-linked and α2,6-linked sialic acid in solution.

The STD NMR analysis of HPyV12 VP1 reveals that the protein interacts with α2,3- and α2,6-linked Neu5Ac (represented by 3′SL and 6′SL, respectively) in solution. In both cases, the interactions involve only the terminal Neu5Ac moiety common to both glycans, as no resonances for protons from Gal and Glc are observed (Fig. 2E to H). Again, these observations are in excellent agreement with the HPyV12 crystal structure, which shows that the Neu5Ac moiety of 3′SLN mediates contacts with VP1 while the remaining portion of the glycan faces into solution and does not participate in any interaction with VP1 (Fig. 2C). The binding site could also clearly accommodate α2,6-linked Neu5Ac, for instance in 6′SL.

### Location of NJPyV and HPyV12 binding sites on VP1.

**FIG 3:**
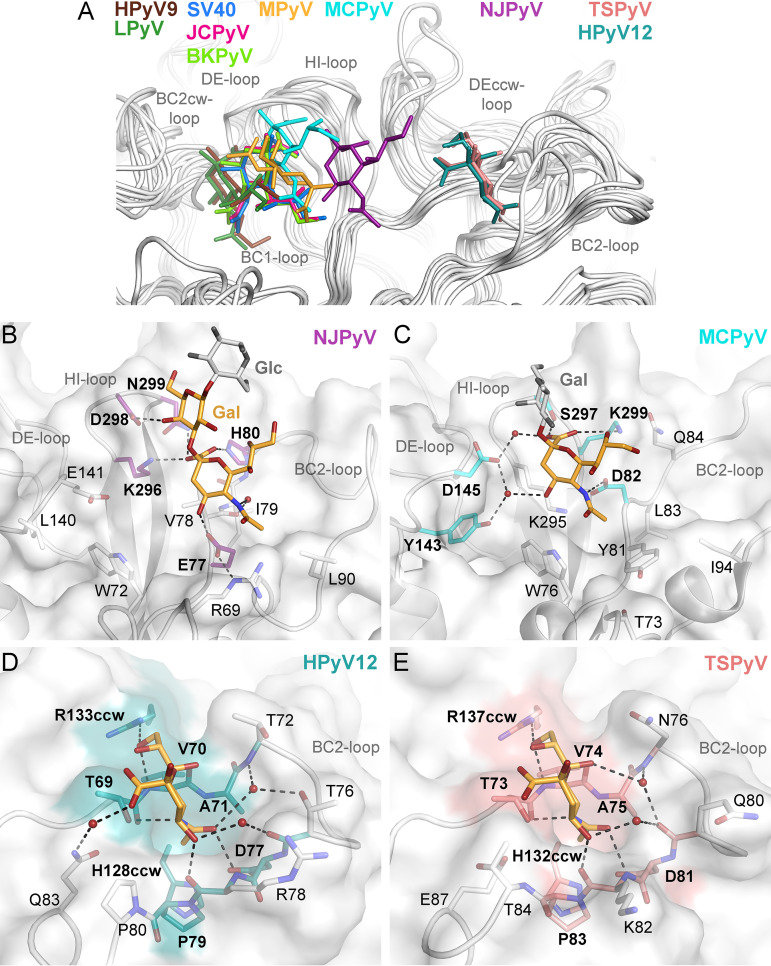
Glycan receptor-binding sites from HPyV12 and NJPyV compared to those from other structurally characterized polyomaviruses. (A) Structural superposition of polyomavirus VP1-glycan complex structures: HPyV12 VP1-3′SLN, NJPyV VP1-3′SL, SV40 VP1-GM1 glycan (PDB 3BWR), BKPyV VP1-GD3 glycan (PDB 4MJ0), JCPyV VP1-LSTc (PDB 3NXD), MCPyV VP1-GD1a glycan (PDB 4FMJ), LPyV VP1-3′SL (PDB 4MBY), MPyV VP1–Neu5Ac-α2,3-Gal-β1,3-[α2,6-Neu5Ac]-GlcNAc-β1,3-Gal-β1,4-Glc (PDB 1VPS), HPyV9 VP1–3′ Neu5Gc-SL (PDB 4POT), and TSPyV VP1-GM1 glycan (PDB 4U60). In the case of structures with more-complex glycans, only the Neu5Ac residues are shown. (B and C) Comparison of binding sites from NJPyV and MCPyV. Glycan residues not involved in intermolecular interactions are colored in gray. Direct and water-mediated hydrogen bonds between VP1 side chain residues and the terminal Neu5Ac are depicted as dashed black lines. Side chains of VP1 residues interacting with the glycan via hydrogen bonding or charged interactions are colored in purple and cyan for NJPyV and MCPyV, respectively. (D and E) Binding sites of HPyV12 and TSPyV VP1 are shown in the same orientation. VP1 complex structures were aligned using Cα atoms and the secondary-structure matching (SSM) tool in Coot (76). Only terminal Neu5Ac residues are shown as orange sticks, with carbons in orange, oxygens in red, and nitrogens in blue. Direct and water-mediated hydrogen bonds between VP1 side chain residues and Neu5Ac are depicted as dashed black lines. Conserved VP1 residues are labeled in bold and are colored on the VP1 surface.

In the NJPyV VP1-3′SL complex, the Neu5Ac ring is positioned roughly in between the locations seen for JCPyV VP1 and TSPyV VP1 (Fig. 3A), defining a novel polyomavirus Neu5Ac-binding site. The NJPyV VP1 sialic acid binding mode shares some similarity with MCPyV VP1 binding of sialylated glycans (Fig. 3B and C). Both proteins bind Neu5Ac in an almost identical orientation, although clearly not in the same location. In particular, the *N*-acetyl and carboxylate groups of Neu5Ac maintain multiple directed and nondirected interactions with VP1 of either virus, while the Neu5Ac glycerol chains project away from the respective protein surfaces. Despite the similar orientations, specific interactions are realized by nonhomologous amino acids as the NJPyV sialic acid-binding site is shifted toward the BC2 loop by about 5 Å (Fig. 3B and C). The interactions used by HPyV12 to engage terminal Neu5Ac are strikingly similar to those used by TSPyV (25) (Fig. 3A, D, and E). Superposition of the two complexes based on the VP1 chains brings the bound Neu5Ac moieties into almost perfect congruence (Fig. 3A). Moreover, key residues on both sides of the binding pockets are conserved, and the BC2 loop folds in a very similar manner on top of both VP1 proteins. Therefore, it is not surprising that the direct VP1-Neu5Ac interactions are also conserved between the two viruses (Fig. 3D and E), which share an overall VP1 amino acid sequence identity of 60%.

### NJPyV and HPyV12 share basic glycan receptor-binding site characteristics with related nonhuman viruses.

**FIG 4:**
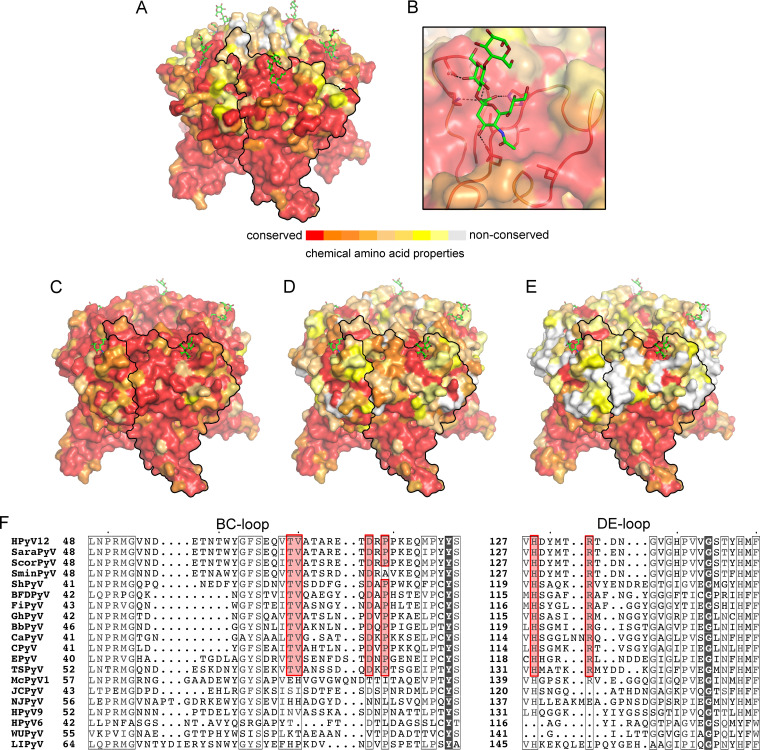
Features of the NJPyV and HPyV12 VP1-Neu5Ac binding sites are conserved in closely related polyomaviruses from different species. Glycan residues are show as sticks, with carbons in green, oxygens in red, and nitrogens in blue. (A and B) VP1 amino acid conservation between NJPyV, ChPyV-Azzi (GenBank accession no. FR692336), ChPyV-Bob (FR692334), ChPyV-Tanu (FR692335), VmPyV1 (NC_019844), and PrufPyV1 (JX159984) is mapped according to the conservation of chemical amino acid properties onto the NJPyV VP1-3′SL complex structure shown in overall and close-up views. (C) Based on the HPyV12 VP1-3′SLN complex structure, amino acid conservation between HPyV12 and shrew polyomaviruses *Sorex minutus* polyomavirus (SminPyV) (MF401583, MF624713, MF624714), *Sorex coronatus* polyomavirus (ScorPyV) (MF374999, MF375000, MF375001, MF401583), and *Sorex araneus* polyomavirus (SaraPyV) (MF374995, MF374996, MF374997) is shown. (D and E) VP1 amino acid conservation between HPyV12 and ShPyV (AKC98332.1) (D) and HPyV12 and three representative avian polyomaviruses, finch polyomavirus (FiPyV; NC_007923), goose hemorrhagic polyomavirus (GhPyV; AEC12236.1), and budgerigar fledgling disease polyomavirus (BFDPyV) (AY672646) (E), are mapped onto the HPyV12 VP1-3′SLN complex structure. (F) Structure-based sequence alignment of BC2 and DE loop VP1 regions from HPyV12, avian, and representative other polymaviruses, namely SaraPyV (MF374995), SminPyV (MF401583), canary polyomavirus (CaPyV; NC_017085), butcherbird polyomavirus (BbPyV; NC_023008), crow polyomavirus (CPyV; NC_007922), equine polyomavirus (EPyV; NC_017982), TSPyV (YP_003800006.1), McPyV1 (AXS76441.1) JCPyV (NP_043511.1), HPyV9 (YP_004243705.1), HPyV6 (ADE45444.1), WU polyomavirus (WUPyV; ARX17335.1), and Lyon IARC PyV (LIPyV; KY404016). Conserved key residues of the BC2 loop Neu5Ac binding site are highlighted in red. NCBI GenBank numbers are given in parentheses.

Phylogenetic analysis of the polyomavirus family based on the complete viral genome revealed that many human polyomaviruses are closely related to one or more simian polyomaviruses (10). These relationships are also present on the amino acid level of VP1. NJPyV shares amino acid sequence identities of about 84% with ChPyV isolates (ChPyV-Azzi, ChPyV-Bob, and ChPyV-Tanu) and about 73% with two other primate polyomaviruses, vervet monkey polyomavirus 1 (VmPyV1) and *Piliocolobus rufomitratus* polyomavirus 1 (PrufPyV1). All of these viruses belong to the *Alphapolyomavirus* genus. In contrast, the phylogenetic classification of HPyV12 is not very clear. A phylogenetic relationship to ShPyV with a VP1 amino acid sequence identity of 60% has been proposed (2), but also VP1 proteins of viruses from the distinct *Gammapolyomavirus* clade comprising the avipolyomaviruses GhPyV and BFDPyV and also FiPyV, crow polyomavirus (CPyV), butcherbird polyomavirus (BbPyV), and canary polyomavirus (CaPyV) exhibit amino acid sequence identities of about 58% with HPyV12 VP1. Moreover, a recent study found full-genome sequence identities of more than 86% between HPyV12 and shrew polyomaviruses and questioned if HPyV12 is a truly human-tropic virus (40). In order to rationalize the impact of VP1 differences on receptor specificity of closely related viruses from different species, we mapped amino acid differences on the surfaces of the NJPyV and HPyV12 VP1 pentamers (Fig. 4). Differences map predominantly to the exposed surface loops on top of VP1. Interestingly, the elongated DE loop, a prominent feature of NJPyV VP1, is unique to this human virus, whereas VP1 residues involved in the recognition of the Neu5Ac-Gal motif are highly conserved in ChPyV, VmPyV1, and PrufPyV1 (Fig. 4A and B). The Neu5Ac-binding region of HPyV12 VP1, formed primarily by the BC2 loop, is as expected mostly conserved, as is the rest of the VP1 surface for the shrew polyomaviruses Sorex coronatus polyomavirus 1 (ScorPyV1) and Sorex araneus polyomavirus 1 (SaraPyV1) (Fig. 4C and F). However, residues P79 and P80 at the base of the Neu5Ac binding site are not present in the shrew Sorex minutus polyomavirus 1 (SminPyV1) isolates (40). Furthermore, residues important for Neu5Ac recognition are conserved not only in ShPyV VP1 (Fig. 4D) but also in the avian virus sequences, whereas most of the VP1 top surface differs (Fig. 4E). A broader VP1 sequence alignment reveals that six key residues of the BC2 and DE loop Neu5Ac-binding region (T69, V70, D77, P79, H128, and R133 in the case of HPyV12) are indeed strictly conserved across the avipolyomaviruses, ShPyV, EPyV, and TSPyV, in contrast to the rest of the residues at the VP1 surface (Fig. 4F). In contrast, these key residues are not conserved in a recently discovered animal polyomavirus from nutria (Myocastor coypus polyomavirus 1 [McoyPyV1]), which clusters in the phylogenetic tree based on the complete amino acid LTAg region (41).

Nevertheless, predictions of the surface loop architecture can be rather error-prone, especially with the low sequence conservation in the case of HPyV12, hampering modeling and mapping of glycan binding sites and the prediction of glycan receptor specificities based only on sequence information. Hence, we used two different approaches to test our hypothesis of the existence and conservation of the core Neu5Ac-binding sites of NJPyV and HPyV12 in the respective viruses. In both cases, we expressed and purified VP1 proteins of closely related nonhuman viruses, specifically, ChPyV for the comparison with NJPyV and ShPyV, GhPyV, and FiPyV for the comparison with HPyV12.

### Interactions of ChPyV with Neu5Ac.

**FIG 5:**
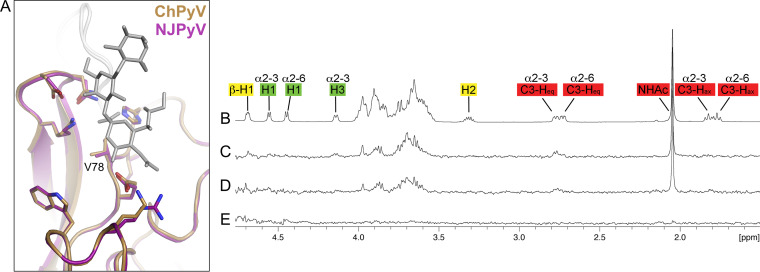
STD NMR spectroscopy of ChPyV VP1 and a putative Neu5Ac-negative ChPyV VP1 mutant. (A) Superposition of NJPyV VP1-3′SL (pink) and native ChPyV VP1 (gold) structures in cartoon representation. NJPyV VP1 side chains responsible for binding of 3′SL and respective ChPy VP1 residues are shown as sticks, with oxygen and nitrogen atoms colored in red and blue, respectively. (B) 1H NMR reference spectrum of 27 μM NJPyV VP1 with a mix of 1 mM 6′SL and 1 mM 3′SL. (C) STD NMR difference spectrum of the same sample as shown in panel B. (D) STD NMR difference spectrum of 50 μM ChPyV VP1 with the 1 mM 6′SL–1 mM 3′SL mix. (E) STD NMR difference spectrum of 50 μM ChPyV VP1 V78F with the 1 mM 6′SL–1 mM 3′SL mix.

As the Neu5Ac-binding site residues of NJPyV are highly conserved in ChPyV VP1, we hypothesized that this virus is also able to bind 3′SL. In order to define the ligand binding properties of ChPyV, we solved the unliganded structure of its VP1 pentamer at 1.9 Å resolution (see Table S2 in the supplemental material). With an RMSD of about 0.5 Å, the ChPyV VP1 monomer superposes very well with a VP1 monomer of the NJPyV VP1-3′SL complex structure, including the region that binds sialic acid in NJPyV (Fig. 5A). The putative ligand-binding sites are largely blocked in the ChPyV VP1 crystals, preventing soaking with ligands. Cocrystallization with sialylated glycans did not yield well-diffracting crystals. Therefore, we assessed the binding of ChPyV VP1 to 3′SL by STD NMR spectroscopy and introduced a valine-to-phenylalanine mutation (V78F) (equivalent to amino acid residue V78 of NJPyV) in the putative sialic acid-binding site of ChPyV in order to interfere with potential glycan binding (Fig. 5B to E). While the STD NMR difference spectrum of wild-type ChPyV VP1 closely resembles that obtained for NJPyV VP1 (Fig. 5C and D), no saturation transfer was observed for the V78F mutant (Fig. 5E). We therefore conclude that ChPyV binds 3′SL in a manner that is identical to that observed for NJPyV. Hence, from our receptor-focused point of view, the two viruses can indeed be regarded as a closely related “human-simian pair.”

### Structures of VP1 proteins of the animal viruses ShPyV, GhPyV, and FiPyV.

Our structure-based sequence analysis shows that the BC2 loop of HPyV12, which essentially mediates all interactions with Neu5Ac, is especially highly conserved in several animal viruses (Fig. 4). In order to probe whether these viruses are able to bind Neu5Ac in a similar manner, and whether differences in regions surrounding the binding site might contribute to subtle differences in receptor engagement, we investigated the structures and ligand binding properties of three representative animal viruses from this alignment: the mammalian ShPyV, which was found in commercial sheep meat (2), and two avian polyomaviruses infecting geese (GhPyV) and finches FiPyV (39). GhPyV is a bona fide animal pathogen, as it causes hemorrhagic nephritis enteritis in geese (59).

**FIG 6:**
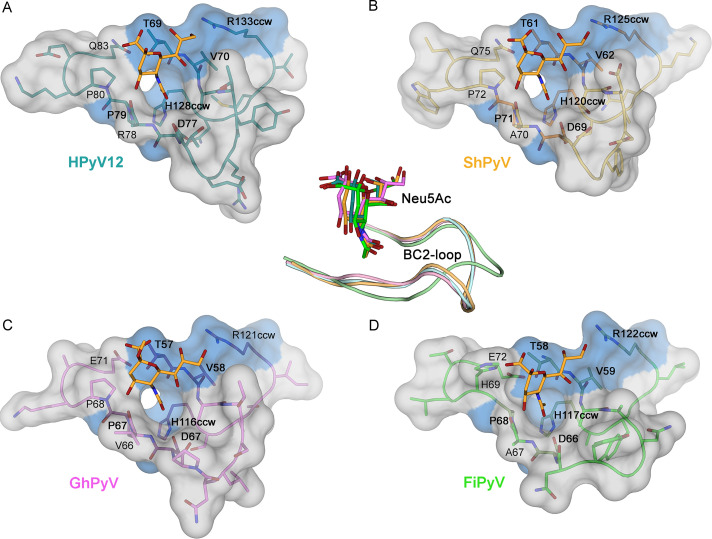
Engagement of Neu5Ac in a highly conserved binding site. Illustration of the BC2 loop binding sites from the complex structures of HPyV12 VP1-3′SLN (A), ShPyV VP1-6′SLN (B), GhPyV VP1–2-*O*-Me-Neu5Ac (C), and FiPyV VP1–2-*O*-Me-Neu5Ac (D) in the same orientation. Only the terminal Neu5Ac portions of the respective glycan structures are shown. Strictly conserved protein residues are distinguished by a blue surface. The middle panel displays a structural superposition of the four binding modes with the Neu5Ac coloring according to panels A to D.

We expressed and purified assembly-incompetent VP1 pentamers from all three viruses by following the protocol established for HPyV12 and solved their crystal structures at high resolution (Table S1). All three proteins fold into the familiar VP1 pentamer structure (53). Superpositions of the VP1 pentamers of ShPyV, GhPyV, and FiPyV with that of HPyV12 confirm that the core VP1 structure is highly conserved (RMSD values of 0.65 Å, 0.83 Å, and 0.73 Å, respectively) (55). There are subtle differences in surface loop regions, however. In HPyV12 VP1, the BC2 loop comprising the Neu5Ac-binding site is formed by amino acids T69 to Q83 (T73 to E87 in TSPyV VP1), whereas the putative binding sites in ShPyV VP1, GhPyV VP1, and FiPyV VP1 include amino acid residues T61 to Q75, T57 to E71, and T58 to E72, respectively (Fig. 6).

10.1128/mBio.00745-20.2 TABLE S1 Crystallographic data collection and refinement statistics for the complexed ShPyV, GhPyV, and FiPyV VP1 structures. Download Table S1, DOCX file, 0.02 MB. Copyright © 2020 Ströh et al. 2020 Ströh et al. This content is distributed under the terms of the Creative Commons Attribution 4.0 International license.

10.1128/mBio.00745-20.3 TABLE S2 Crystallographic data collection and refinement statistics for the native ShPyV, GhPyV, FiPyV, and ChPyV VP1 structures. Download Table S2, DOCX file, 0.01 MB. Copyright © 2020 Ströh et al. 2020 Ströh et al. This content is distributed under the terms of the Creative Commons Attribution 4.0 International license.

### Complexes of ShPyV, GhPyV, and FiPyV VP1 with glycans.

In order to define the ligand binding properties of the three animal viruses ShPyV, GhPyV, and FiPyV, we conducted crystal soaking experiments with sialylated glycans, which yielded additional electron density for the glycan ligand (Fig. S1) so that we could solve and refine complex structures for ShPyV VP1 with 6′SLN and 3′SLN, GhPyV VP1 with 2*-O*-Me-Neu5Ac, and FiPyV VP1 with 2-*O*-Me-Neu5Ac to 1.6 Å, 1.65 Å, 1.95 Å, and 2.8 Å resolution, respectively (Table S1).

10.1128/mBio.00745-20.1 FIG S1 Representative simulated annealing difference electron density maps contoured at a σ level of 2.5 are displayed with a radius of 1.8 Å around the respective ligand in the ShPyV VP1-6´SLN (A), ShPyV VP1-3´SLN (B), GhPyV VP1–2-*O-*Me-Neu5Ac (C), and FiPyV VP1–2-*O*-Me-Neu5Ac (D) complex structures. Download FIG S1, TIF file, 2.5 MB. Copyright © 2020 Ströh et al. 2020 Ströh et al. This content is distributed under the terms of the Creative Commons Attribution 4.0 International license.

All three proteins bind Neu5Ac in a shallow, conserved binding site above the BC2 loop (Fig. 6). The location of this site on the protein and the contacts are highly similar to those of HPyV12 VP1 (Fig. 6) and TSPyV. In fact, the site was first identified and shown to be functionally relevant in TSPyV (25), a human virus. Although a range of different sialyloligosaccharides (such as α2,3- and α2,6-linked sialyllactosamine) were used to generate complexes, the complex structures show that only Neu5Ac forms interactions with the four proteins. The residues that mediate interactions with Neu5Ac are highly conserved among the four proteins.

In the case of ShPyV VP1, the Neu5Ac *N-*acetyl methyl group is situated in a shallow groove in the BC2 loop formed by residues T61, V62, S63, D69, A70, P71, P72, and H120ccw (Fig. 6B). Neu5Ac binding further involves the side chain of residue T61, which forms a hydrogen bond with the *N*-acetyl group amide. The *N*-acetyl carbonyl oxygen interacts with the backbone amide and the Neu5Ac C-4 hydroxyl group interacts with the backbone carbonyl oxygen of residue A70. The ligand’s glycerol moiety interacts with the R125ccw side chain, forming a hydrogen bond between the Neu5Ac C-8 hydroxyl group and a guanidinium N of the arginine. In two of the binding sites, an additional interaction between the Neu5Ac C-9 hydroxyl group and the side chain of R131ccw is formed (not shown in Fig. 6B). The Neu5Ac carboxylate forms a hydrogen bond with the side chain oxygen of residue Q75. Additional carbohydrate monomers of the trisaccharide 6′SLN could be built into the electron density at four equivalent sites in other VP1 chains, showing electron density for Gal in three sites and for Gal-GlcNAc (lactosamine) in one site, respectively. These additional moieties do not, however, form any interactions with the protein and are most likely visible due to reduced glycosidic linkage flexibility in the context of crystal contacts. In the complex structure of ShPyV VP1 and 3′SLN, additional electron density allowed modeling of Gal in four of the six binding sites occupied by Neu5Ac; however, these galactose moieties do not contribute to the overall binding of the glycan to the protein. The interactions between the protein and the Neu5Ac part of 3′SLN are identical to the interactions observed in the 6′SLN complex structure.

GhPyV VP1 basically undergoes the same interactions with its glycan receptor, which in this case is represented by 2-*O*-Me-Neu5Ac (Fig. 6C). The shallow groove in the BC2 loop is formed by residues T57, V58, A59, D65, V66, P67, P68, and H116ccw. Again, residue T57 interacts with the Neu5Ac *N*-acetyl amide, and the backbone amide and carbonyl oxygen of residue V66 interact with the *N-*acetyl carbonyl oxygen and the Neu5Ac C-4 hydroxyl group, respectively. The carboxylate interacts with E71 via a hydrogen bond. The glycerol chain C-8 hydroxyl group forms a hydrogen bond with the side chain of R121ccw.

FiPyV VP1, which was also derivatized with 2-*O*-Me-Neu5Ac, forms interactions with Neu5Ac that are very similar to those observed in GhPyV and ShPyV VP1 (Fig. 6D). The methyl group of the *N-*acetyl moiety reaches into a shallow groove formed by residues T58, V59, A60, D66, A67, P68, E72, and H117ccw. The *N*-acetyl amide is recognized by the hydroxyl group of T58. VP1 residue A67 interacts with the Neu5Ac carbonyl oxygen and C-4 hydroxyl via its backbone atoms, as described above for ShPyV VP1. The R122ccw side chain interacts with the Neu5Ac glycerol moiety, contacting the hydroxyl groups of C-8 or C-9, depending on the VP1 chain. Residue H69 of the FiPyV VP1 contacts both the Neu5Ac carboxylate group and the E72 side chain, thereby providing for an additional hydrogen bond between the protein and the putative Neu5Ac-containing receptor.

In order to obtain insights into possible determinants of specificity that extend beyond direct contacts with Neu5Ac, we analyzed the structures and amino acid compositions of the four BC2 loop sequences in more detail. Structurally, the Neu5Ac-binding regions superimpose well. We calculated all-atom RMSD values with PyMOL using the BC2 loop of HPyV12 VP1 as a reference. With a value of 1.82 Å, the BC2 loop of FiPyV VP1 shows the largest deviation, while the BC2 loops of ShPyV and GhPyV VP1 yield lower deviations, of 0.77 Å and 0.84 Å, respectively. However, the observed structural conservation of the BC2 loop translates only partially to a conservation of residues surrounding the Neu5Ac-binding site. While the residues that mediate direct contacts with Neu5Ac (and are thus determinants of specificity for this portion of the receptor) are highly conserved, the remaining residues, even in the direct vicinity of Neu5Ac, display a much lower level of conservation (Fig. 4F).

### Putative recognition of Neu5Gc by human and animal VP1.

**FIG 7:**
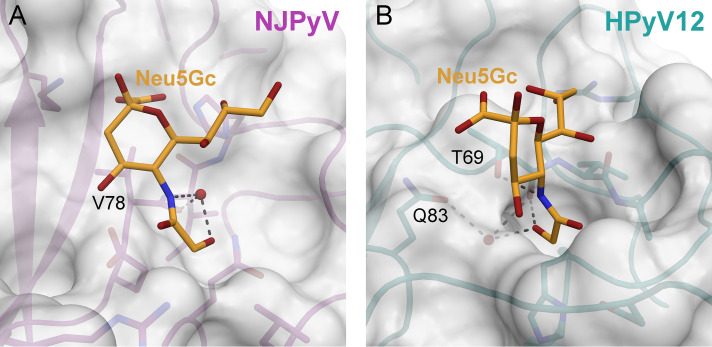
Models of putative Neu5Gc binding in NJPyV and HPyV12 VP1. Neu5Gc models are shown as sticks with carbons colored in orange, oxygens in red, and nitrogens in blue. NJPyV VP1 (A) and HPyV12 VP1 (B) are shown as cartoons with a transparent surface representation around the proteins. Putative hydrogen bonds involving the *N*-glycolyl group of Neu5Gc are depicted as dashed lines.

Humans lack *N*-glycolylneuraminic acid (Neu5Gc) in their receptors due to a loss of the CMP-*N*-acetylneuraminic acid hydroxylase (CMAH) gene activity (60). However, Neu5Gc is a common component of cell surface glycans in many mammals, including nonhuman primates. In the case of the simian polyomavirus SV40, it could be demonstrated that ganglioside Neu5Gc-GM1 is a better receptor than its Neu5Ac counterpart (30), whereas the closely related human BK polyomavirus is unable to engage Neu5Gc-containing receptors (24). The human polyomavirus 9 (HPyV9) and the related B-lymphotropic polyomavirus (LPyV) of the African green monkey have counterintuitive binding properties, as the human virus displays a preference for the Neu5Gc receptor variants (22, 24). In order to assess the Neu5Gc binding capacities of the VP1 proteins introduced in this study, we prepared representative models of the Neu5Gc complexes of NJPyV und HPyV12 VP1 by replacing the Neu5Ac moiety with a Neu5Gc model within the complex X-ray structures (Fig. 7). These models demonstrate that the sialic acid-binding sites of both NJPyV and HPyV12 VP1 can accommodate Neu5Gc. In the NJPyV VP1 complex, the additional hydroxyl group of the *N*-glycolyl group would be able to establish a hydrogen bond with the water molecule that bridges the Neu5Gc amide nitrogen and the protein backbone at residue V78 (Fig. 7A). In HPyV12 VP1, the Neu5Ac-binding site possesses a recessed cavity with a hydrophilic bottom side that potentially prefers the extra hydroxyl group of Neu5Gc. Here, the hydroxyl could establish a hydrogen bond with a water molecule held by the side chain of Q83 and the backbone of T69 (Fig. 7A). The animal PyV species of chimpanzee and sheep, goose, and finch possess highly conserved binding pockets (Fig. 5A and 6, respectively) compared with their related human VP1 counterparts, and they therefore should be able to engage Neu5Gc as well. Mutations within the core binding sites, which would reflect evolutionary changes to preferentially accommodate Neu5Gc over Neu5Ac in the case of the animal PyVs, were not detected. However, Neu5Gc-favoring amino acid changes with rather long-range effects cannot be ruled out.

## DISCUSSION

We solved the crystal structures of the VP1 proteins of the recently discovered polyomaviruses NJPyV and HPyV12 and established that the two viruses use significantly different binding site architectures to bind sialylated glycans. The NJPyV structure defines a new sialic acid-binding site in polyomaviruses, while HPyV12 uses a site that was previously observed in the human TSPyV. In each case, however, the respective binding mode is conserved in closely related animal polyomaviruses. For NJPyV, this is demonstrated in solution by STD NMR spectroscopy of 3′SL binding to ChPyV VP1 and by the structure determination of unliganded ChPyV VP1. For HPyV12, we have solved crystal structures of three animal polyomavirus VP1 proteins (ShPyV, GhPyV, and FiPyV) that show essentially the same interactions and specificity for a terminal sialic acid. These results advance an understanding of virus-glycan specificities, and they also provide insight into the evolution of polyomaviruses.

Although many polyomaviruses engage sialylated receptors, extensive structure-function analyses have shown that there is remarkable plasticity in the recognition of sialic acids. The previously reported structures of polyomavirus complexes identified no less than five different binding modes of sialic acid. One is exemplified by JCPyV (21), BKPyV (14), and SV40 (15), the second by murine polyomavirus (26), a third by MCPyV (23), a fourth by LPyV (22) and HPyV9 (24), and a fifth by TSPyV (25). The five binding sites differ not only in their location on VP1 but also in the orientation of the bound sialic acid and the individual interactions that confer glycan specificity. The structure of NJPyV in complex with 3′SL extends this level of plasticity even further with yet another sialic acid-binding site that lies in between the sites observed in other polyomaviruses and that shares some similarities with respect to individual contacts with MCPyV. A common feature of the Neu5Ac binding sites of SV40, JCPyV, BKPyV, LPyV, and HPyV9 is a hydrophobic pocket, which is formed by residues of HI and BC1 loops from one VP1 monomer and BC2 loop residues of the clockwise (cw) VP1 monomer (14, 15, 21, 22, 24). The location of this hydrophobic pocket is not conserved in NJPyV VP1. Instead, BC2 loop residues E77 and L90 provide a similar hydrophobic environment for the methyl group of Neu5Ac on NJPyV VP1 in a different location.

The plasticity of polyomavirus-sialic acid interactions outlined in this and previous reports is, at least to our knowledge, unprecedented. While many viruses recognize sialylated glycans, they typically do so with a single binding site that is conserved across strains. A well-documented example is the influenza virus hemagglutinin (HA)-sialic acid interaction (61). While human and avian HAs bind to sialylated glycans that differ markedly in linkage type and overall conformation, all HA proteins essentially recognize the terminal sialic acid of the sialoglycan receptors in the same region and bind Neu5Ac in the same orientation (62). The variety of sialic acid-binding sites seen in polyomaviruses is particularly striking, as even identical glycans (such as 3′SLN) are bound in different locations by different polyomaviruses. In these cases, the viruses have evolved differing strategies to bind not only the Neu5Ac itself but also the glycosidic linker region, thus achieving specificity for one ligand with entirely different contacts. It is possible that this is a consequence of the actual physiological glycan receptors having more-complex structures that are not reflected by 3′SLN, perhaps branched glycans that necessitate binding in different orientations to either virus. Another possibility is that fine-tuning of affinity to a specific ligand can be realized only in a certain location.

The structural analyses of the HPyV12, ShPyV, GhPyV, and FiPyV VP1 proteins establish the existence of essentially the same sialic acid-binding site in viruses that infect different hosts. The same site also exists in the human-tropic TSPyV VP1, where it was shown to be required for cell attachment and infection (25). In all five cases, interactions with Neu5Ac are limited to a small set of highly conserved residues, and these residues are clearly sufficient to confer specificity for Neu5Ac. In contrast, the residues surrounding the Neu5Ac-binding site display a much lower level of conservation. One possible explanation is that there is no evolutionary pressure on the virus capsids to maintain conservation that goes beyond direct interactions with Neu5Ac because this pyranose is the sole binding determinant. However, given the narrow receptor specificities observed for other polyomaviruses, this seems unlikely: all other known polyomavirus-sialyloligosaccharide complexes show interactions that extend beyond the Neu5Ac ring, and it is precisely these additional interactions that determine (in some cases extraordinary) specificity for a particular sialylated glycan or a small range of structurally related glycans (12, 14, 15, 21–24). Another explanation is that TSPyV, HPyV12, ShPyV, GhPyV, and FiPyV utilize sialic acid-based receptors that contain sialic acid variants other than Neu5Ac. Interestingly, the BC2 loop binding cavity features a hydrophilic bottom and would potentially prefer the extra hydroxyl group of Neu5Gc. However, Neu5Gc-favorable mutations within the core binding sites, which would suggest preferential binding of Neu5Gc over Neu5Ac in case of the animal PyVs, were not detected.

Certainly, it is conceivable that the residues surrounding the sialic acid-binding site contribute to the host tropism, perhaps by providing contacts with sialic acid variants or linkages that are preferentially expressed in a certain host and/or a certain tissue. In addition, the hemagglutinating activity of polyomaviruses has been attributed primarily to the binding of VP1 to sialylated glycans on the surface of human red blood cells (63–65). Conversely, the absence of hemagglutination activity for a given virus does not preclude glycan receptor binding, as shown for SV40 and its specific sialylated receptor, the GM1 ganglioside (13, 15). In the case of avian viruses BFDPyV, CaPyV, and GhPyV, hemagglutination activity has been reported with chicken and human erythrocytes, functionally supporting the sialylated glycan receptor binding properties we report for GhPyV (66, 67). However, FiPyV did not hemagglutinate chicken or human erythrocytes (66), suggesting an alternative receptor specificity compared to that of the related viruses, likely influenced by residues surrounding the conserved BC2 loop Neu5Ac-binding site.

Additionally, unknown nonsialylated coreceptors may likewise impact the host tropism.

The identification of shrew polyomaviruses (40) and McoyPyV1 in nutria (41), which are closely related in sequence to HPyV12, and studies reporting low seroprevalence rates and seroactivity for HPyV12, NJPyV, and LIPyV questioned the tropism of recently discovered human polyomaviruses (36). Further analyses are needed, but the presence of a functional core BC2 loop sialic acid-binding site in TSPyV nonetheless demonstrates the conservation of this site in a human-tropic virus (25).

Past studies suggest coevolution of polyomaviruses with their hosts (2, 68) and exclude host-switching events between members of distantly related species. Nevertheless, ancient or sporadic viral transmission events between members of more closely related species or subspecies have to be considered. However, virus-receptor interactions and their impact on transmissibility and zoonotic events often cannot be broken down to a single parameter, as shown for the prominent example influenza A virus and its glycan receptor (69, 70). The interplay of HA-glycan receptor specificity, affinity, and avidity as well as conformational glycan flexibility and HA glycosylation may lead to pandemics if these are sufficient to allow barrier crossovers such as animal-to-human transmission, virus-cell interaction, and finally human-to-human transmission (69).

In conclusion, our data provide clear evidence for close relationships between human and animal polyomaviruses. Such relationships have been suggested by sequence analyses (2, 38, 39, 47), but as the polyomavirus VP1 and LTAg proteins generally display high levels of sequence conservation, analyses based on sequence alone can be misleading. The structures reported here extend the phylogenetic analyses, demonstrating that key properties of such human-animal pairs, most importantly the engagement of sialic acid receptors, are highly conserved even between distantly related viruses. We expect that this detailed analysis will provide a foundation for assessing receptor-based cell tropism changes and cross-species transmission events, and it may also help to advance an understanding of the evolutionary history of polyomaviruses and perhaps even other viruses.

## MATERIALS AND METHODS

### Cloning, expression, and protein purification.

The NJPyV VP1 DNA was provided by Nischay Mishra (Columbia University), and the pET15b-derived expression plasmid for NJPyV VP1 (amino acids 36 to 323; NCBI accession no. YP_009030020) was obtained via the InFusion HD cloning strategy according to the manufacturer’s protocol (Clontech). Therefore, the vector was linearized via XhoI and the DNA was amplified using the following primers: fw, 5′-CAGCCATATGCTCGACGGAGGAGTTGAAGTTTTAAATA-3′; rev, 5′-CAGCCGGATCCTCGATTAATTTTTTACAGCCCTTTTTCTC-3′.

A synthetic Escherichia coli codon-optimized gene coding for amino acids 27 to 299 of HPyV12 VP1 (NCBI accession no. YP_007684355) was amplified by PCR and cloned into the pET15b vector (Novagen) via NdeI and BamHI restriction enzymes in-frame with an N-terminal hexahistidine tag (His tag) and a thrombin cleavage site. The following primers were used: fw, 5′-GCATCATATGGGCGGTATTGAGGTTCTGGATGTGAAAAC-3′; and rev, 5′-GACTGGATCCTAGTTACGAACGGCACGTTTGCGC-3′.

The DNA for the E. coli codon-optimized expression constructs for an equivalent N- and C-terminally truncated VP1 of ChPyV (NCBI accession no. YP_004046682.1), including a mutation of the conserved cysteine in the CD loop (C154S), was purchased from Eurofins Genomics, amplified by PCR, and cloned into the NdeI- and BamHI-linearized pET15b vector by use of InFusion HD cloning. The following primers were used: ChPyV VP1 fw, 5′-CGCGCGGCAGCCATATGGGTGGCGTTGAGGTC-3′; and ChPyV VP1 rev, 5′-GTTAGCAGCCGGATCTTAATTCTTCACGGCACGTTTGCG-3′. Site-directed mutagenesis of ChPyV for the STD NMR experiment was carried out using primers as follows (mismatched nucleotides are indicated by boldface): ChPyV V78F fw, 5′-GTACGGTTATAGCGAA**TT**CATTCACCATGCCGATGGGTATG-3′; ChPyV VP1 V78F rev, 5′-CATACCCATCGGCATGGTGAATG**AA**TTCGCTATAACCGTAC-3′.

The E. coli codon-optimized VP1 expression constructs in pET15b (cloning sites NdeI and BamHI) for GhPyV (amino acids 21 to 286; NCBI accession no. AEC12236.1) and ShPyV (amino acids 20 to 285, including a C95S mutation; NCBI accession no. AKC98332.1) were purchased from GeneScript (NJ, USA). For FiPyV VP1, the E. coli codon-optimized construct (amino acids 22 to 291; NCBI accession no. YP_529833) was purchased from General Biosystems, Inc. Due to aggregation and oxidation problems during purification trials of an earlier construct (not shown here), an additional C78S mutation was introduced next to the canonical C92S. Furthermore, the construct contained a TEV protease site following the His tag. It was cloned from NcoI to BamHI into the pET-15b vector. The amino acid numbering of all VP1 proteins excludes the N-terminal methionine.

Expression of NJPyV VP1 in E. coli Rosetta 2 (DE3) and of HPyV12, ChPyV, ChPyV-V78F, ShPyV, GhPyV, and FiPyV VP1 in E. coli BL21(DE3) was induced by addition of 0.4 mM IPTG (isopropyl-β-d-thiogalactopyranoside) and carried out at 20°C for about 18 h. The truncated VP1 cannot assemble into a capsid but forms pentamers or cysteine-mediated dimers of pentamers in solution via the conserved cysteine in the CD loop if present in the expression construct. After cell lysis, VP1 proteins were first purified by nickel affinity chromatography and then by size exclusion chromatography on a Superdex-200 column. The His tag was then removed using thrombin (NJPyV, HPyV12, ChPyV, ShPyV, and GhPyV VP1) or TEV protease (FiPyV VP1) prior to a second nickel affinity chromatography and a final size exclusion chromatography step. A nonnative amino acid sequence, GSHM (or GSHMLD in the case of NJPyV VP1), remains at the N terminus after protease cleavage. Buffers were supplemented with 1 M urea for FiPyV VP1 during the nickel affinity chromatography and TEV protease digestion. After size exclusion chromatography, HPyV12 VP1 pentamers were kept in 20 mM HEPES (pH 7.5), 150 mM NaCl, 20 mM dithiothreitol (DTT). Dimers of NJPyV VP1 pentamers and ChPyV, GhPyV, ShPyV, and FiPyV VP1 pentamers were stored in an equivalent buffer without DTT prior to crystallization.

### Crystallization and data collection.

HPyV12 VP1 was concentrated to 7 mg/ml and crystallized at 20°C by hanging-drop vapor diffusion against a reservoir solution containing 4% (vol/vol) Tacsimate (pH 7.0) and 16% (wt/vol) polyethylene glycol (PEG) 3350. These crystals were not suitable for crystal soaking experiments, because all five binding sites of VP1 were blocked in the crystal by symmetry-related copies of VP1. For complex formation, crystals of HPyV12 VP1 were grown by hanging-drop vapor diffusion at 20°C using a protein concentration of 6 mg/ml and a reservoir solution containing 0.2 M ammonium acetate, 0.1 M bis-Tris (pH 7.0), and 45% (vol/vol) 2-methyl-2,4-pentanediol (MPD). These crystals were soaked for 24 h in the reservoir solution supplemented with 20 mM 3′SLN (Carbosynth, UK).

Crystals of NJPyV VP1 obtained at 4°C using the sitting-drop vapor diffusion method, from a reservoir solution containing 0.1 M succinic acid (pH 7.0) and 15% (wt/vol) PEG 3350, and a protein solution with a concentration of 7 mg/ml yielded the native structure of the protein. Crystals grown using the sitting-drop vapor diffusion method in 0.1 M MES (pH 6.5), 12% (wt/vol) PEG 20000 at 20°C were derivatized by incubation for 10 min in a reservoir solution supplemented with 50 mM 3′SL (Carbosynth, UK). For the structure solution of unliganded ChPyV VP1, crystals grew from a protein concentration of 2.8 mg/ml via the sitting-drop vapor diffusion method at 20°C. The crystallization condition included 0.1 M sodium acetate (pH 7.0), 12% (wt/vol) PEG 3350, and 30% (vol/vol) 1,5-diaminopentane dihydrochloride.

The native GhPyV VP1 structure was solved with a crystal grown at 20°C using the sitting-drop vapor diffusion method and a Morpheus screen purchased from Molecular Dimensions, UK (0.9 M nitrate phosphate sulfate [mix of 0.3 M NaNO_3_, 0.3 M Na_2_HPO_4_, 0.3 M (NH_4_)_2_SO_4_, 0.1 M MES/imidazole (pH 6.5), 10% (wt/vol) PEG 8000, 20% (vol/vol) ethylene glycol]. The protein concentration used for crystallization of GhPyV was 3.7 mg/ml. A GhPyV VP1 crystal obtained from the same screen but with a reservoir containing 0.1 M carboxylic acids (0.2 M Na formate, 0.2 M ammonium acetate, 0.2 M Na citrate tribasic, 0.2 M Na oxamate, 0.2 M Na K tartrate), 0.1 M MES/imidazole (pH 6.5), 10% (wt/vol) PEG 8000, and 20% (vol/vol) ethylene glycol was incubated in a drop of reservoir solution supplemented with 20 mM 2-*O*-Me-Neu5Ac for 17 h for complex formation.

ShPyV VP1 was concentrated to 4.1 mg/ml and crystallized at 20°C by the sitting-drop vapor diffusion method. The native structure was obtained from a crystallization setup with a reservoir containing 0.2 M KSCN and 20% (wt/vol) PEG 3350. ShPyV VP1 (3.7 mg/ml) was further crystallized via the hanging-drop vapor diffusion method at 20°C, with a reservoir solution of 0.15 M KSCN and 20% (wt/vol) PEG 3350. These crystals were derivatized in a separate drop supplemented with 20 mM 3′SLN for 30 min. A ShPyV VP1 crystal grown with a reservoir solution containing 0.2 M magnesium chloride, 0.1 M Tris (pH 8.5), and 20% (wt/vol) PEG 800 was transferred into the respective reservoir solution supplemented with 20 mM 6′SLN for 48 h.

FiPyV VP1 was concentrated to 3.7 mg/ml, and the native structure was obtained from a crystal grown at 20°C using the hanging-drop vapor diffusion method. The reservoir solution contained 0.1 M HEPES (pH 7.5), 10% (wt/vol) PEG 8000, and 8% (vol/vol) ethylene glycol. The structure of FiPyV VP1 in complex with 2-*O*-Me-Neu5Ac was solved using a crystal grown at 4°C in a hanging-drop setup with a reservoir containing 0.2 M MgCl_2_, 0.1 M Tris (pH 7), and 10% (wt/vol) PEG 8000. The crystal was soaked in reservoir solution supplemented with 25 mM 2-*O*-Me-Neu5Ac at 4°C for 30 min.

Drops were set up with 1 μl protein solution and 1 μl reservoir solution for HPyV12, ShPyV (3′SLN derivate), and FiPyV VP1 using the hanging-drop method, or 0.4 μl of the protein solution and 0.4 μl of the reservoir solution were used for the crystallization of NJPyV, GhPyV, and ShPyV (apo and 6′SLN derivate) VP1 using the sitting-drop vapor diffusion method. For ChPyV VP1, 0.2 μl protein solution and 0.2 μl reservoir solution were used for crystallization by the sitting-drop vapor diffusion method. All crystals were transferred for about 2 s into the respective reservoir or soaking solution supplemented with 30% (vol/vol) glycerol or 25% (vol/vol) MPD for FiPyV VP1-2-*O*-Me-Neu5Ac and ShPyV VP1-3′SLN prior to freezing in liquid nitrogen. Data sets for HPyV12, ShPyV, GhPyV, FiPyV, and ChPyV VP1 were collected using beamline X06DA (X06SA for native FiPyV VP1) of the Swiss Light Source (SLS), while the data sets for NJPyV VP1 were taken using beamline 14.1 of the Berliner Elektronenspeicherring Gesellschaft für Synchrotronstrahlung (BESSY).

### Structure determination and structural analysis.

Diffraction data sets were processed with XDS (71). All VP1 structures were solved by molecular replacement (MR) using Phaser MR (72) included in the CCP4 program suite (73). The native MCPyV VP1 structure (PDB 4FMG), modified by CHAINSAW (74), was used as a search model in molecular replacement to solve the HPyV12 and NJPyV VP1 structures. The refined NJPyV VP1 structure was used as search model for the structure determination of ChPyV VP1. The refined HPyV12 VP1 pentamer structure was used as a search model to solve the ShPyV VP1 structure, which then served as the search model for the structure determination procedure of GhPyV VP1. The FiPyV VP1 structure was solved using a model of GhPyV VP1. The search models were obtained by truncating the respective structure to the last common atom by CHAINSAW (74). Rigid-body and simulated annealing refinement was carried out with Phenix (75). Subsequently, alternating rounds of model building in Coot (76) and refinement with Refmac5 (77), including 5-fold noncrystallographic symmetry (NCS) restraints, the translation-libration-screw (TLS) method (78), CCP4 library restraints, and user-defined restraints for the α2,3-glycosidic bond in cases where 3′SL or 3′SLN were included as ligands, were performed. In order to investigate patterns of amino acid conservations on the VP1 surface, an alignment was carried out with the multiple sequence alignment tool Clustal Omega (79). JalView (80) was used to assign values for the conservation of the chemical acid amino acid properties: 11 (conserved; red) to 0 (nonconserved; gray). Values of 1 to 0 are colored in gray. For the calculations of RMSD values, Cα atoms of VP1 complex structures were aligned using the secondary-structure matching (SSM) tool in Coot (76). Buried surface and contact areas were calculated with PISA (81).

### STD NMR.

Saturation transfer difference nuclear magnetic resonance (STD NMR) spectra were recorded on a Bruker AVIII-600 MHz spectrometer with a room temperature probe head at temperatures between 285 K and 288 K. Samples were measured in tubes with an inside diameter of 3 mm and contained 27 μM (NJPyV) or 50 μM (HPyV12 and ChPyV mutant and wild type) VP1 and 2 mM of the respective oligosaccharide(s) in 20 mM K_2_HPO_4_/KH_2_PO_4_ (pH 7.4), 150 mM NaCl, 99% D_2_O. Oligosaccharides were purchased from Carbosynth, UK, and resuspended in D_2_O. Data were processed with TOPSPIN 3.0 (Bruker). Off- and on-resonance frequencies of −30 ppm and 7.3 ppm, respectively, were used. The irradiation power and length of the selective pulse train were 57 Hz and 2 s, respectively. A strength of 3.2 kHz was employed to suppress residual protein resonances in a continuous-wave spin-lock pulse. A total of 5,000 scans were recorded, and the relaxation delay was 3 s. Prior to Fourier transformation, spectra were multiplied with a Gaussian window function.

### Data availability.

All coordinates and structure factor amplitudes were deposited into the RCSB Protein Data Bank (https://www.rcsb.org) under the accession numbers shown in parentheses, as follows: NJPyV VP1 (6Y5X), NJPyV VP1-3′SL (6Y5Y), HPyV12 VP1 (6Y5Z), HPyV12 VP1-3′SLN (6Y60), ShPyV VP1 (6Y61), ShPyV VP1-3′SLN (6Y63), ShPyV VP1-6′SLN (6Y64), GhPyV VP1 (6Y65), GhPyV VP1–2-*O*-Me-Neu5Ac (6Y66), FiPyV VP1 (6Y67), FiPyV VP1–2-*O*-Me-Neu5Ac (6Y6A), and ChPyV VP1 (6Y9I). Structure figures were prepared with PyMOL (The PyMOL Molecular Graphics System, version 1.8.0.3; Schrödinger, LLC).
