# Supplementary material for: Structural Basis and Evolution of Glycan Receptor Specificities within the Polyomavirus Family
Source: mBio. 2020 Jul 28;11(4):e00745-20. doi: 10.1128/mBio.00745-20 (PMC7387793; doi:10.1128/mBio.00745-20)
Supplement: TABLE S1 [file mBio.00745-20-st001.docx]

**S1 Table** Crystallographic data collection and refinement statistics for the complexed ShPyV, GhPyV, and FiPyV VP1 structure

|  | **ShPyV VP1-3’SLN** | | **ShPyV VP1-6’SLN** | **GhPyV VP1-2-*O*-Me-Neu5Ac** | **FiPyV VP1-2-*O*-Me-Neu5Ac** |
| --- | --- | --- | --- | --- | --- |
| PDB accession code | 6Y63 | | 6Y64 | 6Y66 | 6Y6A |
| **Data collection** | |  |  |  |  |
| Space group | P2_1_ | | P2_1_ | P1 | P1 |
| a, b, c [Å] | 130.29, 79.93, 145.88 | | 129.62, 81.26, 146.66 | 87.46, 90.54, 101.22 | 86.50, 172.06, 245.62 |
| α, β, γ [°] | 90, 116, 90 | | 90, 115.52, 90 | 94.23, 98.09, 107.87 | 107.09, 97.93, 93.93 |
| Resolution [Å] | 48.81-1.65 (1.75-1.65) | | 47.48-1.60 (1.64-1.60) | 49.58-1.95 (2.00-1.95) | 49.95-2.65 (2.75-2.65) |
| Unique reflections | 322,284 (51,513) | | 350,236 (25,259) | 206,889 (15,085) | 377,841 (36,891) |
| Total reflections | 2,195,448 (244,393) | | 2,372,905 (170,210) | 739,225 (51,908) | 1,019,264 (102,004) |
| R_meas_ [%] | 10.1 (139.7) | | 6.9 (75.7) | 14.6 (79.7) | 27.1 (113.4) |
| I/σI | 13.9 (1.4) | | 17.8 (3.0) | 9.33 (2.0) | 4.6 (1.1) |
| CC_1/2_ [%] | 99.9 (53.6) | | 99.9 (81.1) | 99.3 (68.5) | 97.6 (52.0) |
| Completeness [%] | 99.7 (99.1) | | 96.7 (94.7) | 97.7 (96.4) | 97.9 (96.4) |
| Wilson B-factors [Å^2^] | 29.2 | | 25.4 | 24.6 | 38.4 |
|  |  | |  |  |  |
| **Refinement** | |  |  |  |  |
| R_work_ / R_free_ [%] | 16.9 / 20.0 | | 15.2 / 18.2 | 16.5 / 20.1 | 24.4 / 26.7 |
| No. of atoms | |  |  |  |  |
| Protein | 19,711 | | 19,781 | 20,109 | 78,546 |
| Water | 2,328 | | 2,789 | 2,708 | 2,378 |
| Glycan | 170 | | 226 | 220 | 609 |
| B-factor [Å^2^] | |  |  |  |  |
| Protein | 22.8 | | 19.0 | 17.9 | 37.6 |
| Water | 33.3 | | 30.7 | 27.7 | 21.7 |
| Glycan | 33.8 | | 32.3 | 25.9 | 59.7 |
| R.m.s.d. | |  |  |  |  |
| Bond length [Å] | 0.007 | | 0.009 | 0.008 | 0.006 |
| Bond angles [°] | 1.48 | | 1.51 | 1.47 | 1.34 |
