# Supplementary material for: Structural Basis and Evolution of Glycan Receptor Specificities within the Polyomavirus Family
Source: mBio. 2020 Jul 28;11(4):e00745-20. doi: 10.1128/mBio.00745-20 (PMC7387793; doi:10.1128/mBio.00745-20)
Supplement: TABLE S2 [file mBio.00745-20-st002.docx]

**S2 Table** Crystallographic data collection and refinement statistics for the native ShPyV, GhPyV, FiPyV, and ChPyV VP1 structure

|  | **ShPyV VP1** | **GhPyV VP1** | **FiPyV VP1** | **ChPyV VP1** |
| --- | --- | --- | --- | --- |
| PDB accession code | 6Y61 | 6Y65 | 6Y67 | 6Y9I |
| **Data collection** |  |  |  |  |
| Space group | P3_1_ | P1 | P2_1_ | P1 |
| a, b, c [Å] | 130.43, 130.43, 221.77 | 87.29, 90.41, 100.84 | 145.05, 91.61, 352.33 | 63.41, 82.19, 82.66 |
| α, β, γ [°] | 90, 90, 120 | 94.05, 97.90, 108.06 | 90, 92.10, 90 | 68.61, 77.36, 77.49 |
| Resolution [Å] | 49.77-2.45 (2.51-2.45) | 45.97-1.45 (1.49-1.45) | 48.07-2.62 (2.71-2.62) | 46.46-1.90 (1.95-1.90) |
| Unique reflections | 147,077 (10,067) | 487,470 (34,197) | 274,542 (26,770) | 117,709 (8,633) |
| Total reflections | 1,213,610 (70,550) | 1,744,083 (127,271) | 1,061,205 (99,506) | 817,666 (58,671) |
| R_meas_ [%] | 8.5 (53.5) | 6.7 (72.5) | 26.2 (156.0) | 14.8 (135.2) |
| I/σI | 21.3 (4.9) | 13.8 (2.2) | 6.1 (1.0) | 9.83 (1.61) |
| CC_1/2_ [%] | 99.8 (92.8) | 99.9 (78.8) | 99.3 (64.2) | 99.7 (57.2) |
| Completeness [%] | 94.8 (87.5) | 95.4 (90.5) | 98.2 (94.5) | 99.4 (98.9) |
| Wilson B-factors [Å^2^] | 34.7 | 20.5 | 55.5 | 32.2 |
|  |  |  |  |  |
| **Refinement** |  |  |  |  |
| R_work_ / R_free_ [%] | 16.1 / 19.7 | 14.8 / 17.0 | 27.0 / 30.0 | 17.1 / 21.3 |
| Protein | 20,395 | 20,549 | 58,979 | 10,612 |
| Water | 976 | 3,381 | 751 | 953 |
| Protein | 32.4 | 12.77 | 57.0 | 30.3 |
| Water | 31.9 | 28.01 | 37.8 | 40.23 |
| Bond length [Å] | 0.007 | 0.009 | 0.006 | 0.009 |
| Bond angles [°] | 1.53 | 1.51 | 1.37 | 1.57 |
