# Supplementary figures and images for: Structural Basis and Evolution of Glycan Receptor Specificities within the Polyomavirus Family
Source: mBio. 2020 Jul 28;11(4):e00745-20. doi: 10.1128/mBio.00745-20 (PMC7387793; doi:10.1128/mBio.00745-20)

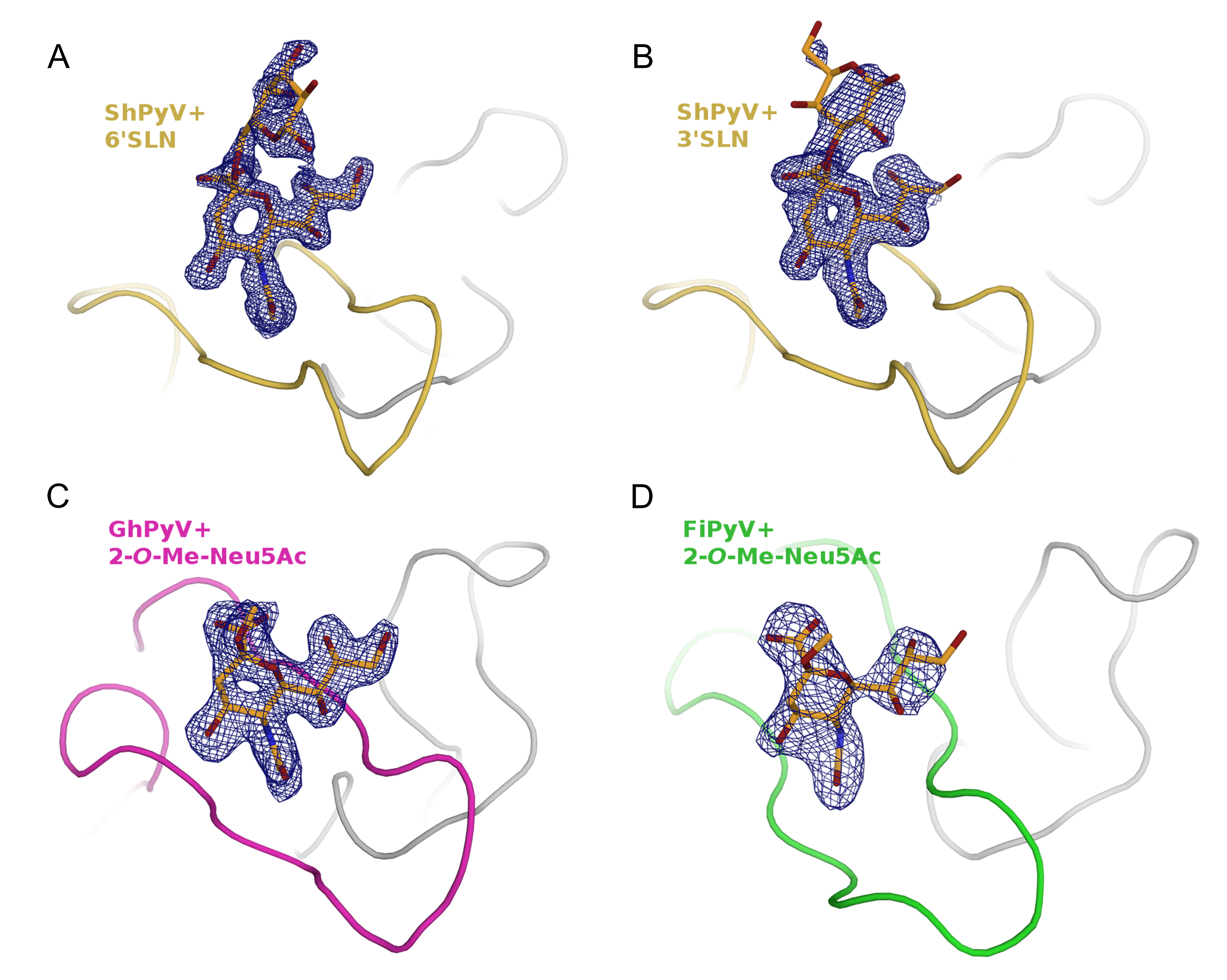

Supplement: FIG S1 [file mBio.00745-20-sf001.tif]
